# EXPERIENCES AND FACILITATORS OF FALLS PREVENTION AMONG ETHNICALLY DIVERSE OLDER ADULTS: A QUALITATIVE STUDY

**DOI:** 10.1093/geroni/igz038.3142

**Published:** 2019-11-08

**Authors:** Ladda Thiamwong, Norma E Conner

**Affiliations:** 1 College of Nursing, University of Central Florida, Orlando, Florida, United States; 2 University of Central Florida, Orlando, Florida, United States

## Abstract

Background: Falls increase as people age and decrease the quality of life. Even though fall interventions have received great attention, fall incidence rates have still arisen. In order for older adults to reap the benefits of evidence-based fall interventions, a challenge of implementation in the real world and right context must be met. Understanding experiences, facilitators, and barriers of fall prevention among four major ethnic groups in the Unites States could be extremely valuable. Objective: The aim of this study was to describe experiences and highlight facilitators and barriers on fall and fear of falling interventions among ethnically diverse community-dwelling older adults. Methods: Four ethnically specified (African American, Asian, Hispanic and Non-Hispanic White) focus groups were conducted. A total of 28 older adults and four family caregivers were interviewed. Interviews covered experiences on falls and fear of falling, attitudes, factors, consequences, risk assessment, and interventions. Data were organized and analyzed with the NViVo software. Results: Falls related experiences and behaviors were multifaceted and varied. Three themes related to falls experiences and behaviors were identified, 1) falls prevention versus fear of falling amplification; 2) role identity, culture and family considerations; and 3) take care of you, take care of me. Facilitators of fall prevention were integration of individual learning within a group meeting, providing appropriate assistive devices and promoting environmental safety. Barriers were inconsistent fall risk assessments, low fall risk awareness and acknowledgment, and balance and visual impairment.

